# Leveraging Positive Psychology to Support Smoking Cessation in Nondaily Smokers Using a Smartphone App: Feasibility and Acceptability Study

**DOI:** 10.2196/13436

**Published:** 2019-07-03

**Authors:** Bettina B Hoeppner, Susanne S Hoeppner, Hannah A Carlon, Giselle K Perez, Eric Helmuth, Christopher W Kahler, John F Kelly

**Affiliations:** 1 Recovery Research Institute Massachusetts General Hospital Harvard Medical School Boston, MA United States; 2 Department of Psychiatry Massachusetts General Hospital Harvard Medical School Boston, MA United States; 3 Center for Anxiety and Traumatic Stress Disorders Massachusetts General Hospital Boston, MA United States; 4 Behavioral Medicine Program Massachusetts General Hospital Harvard Medical School Boston, MA United States; 5 School of Public Health Boston University Boston, MA United States; 6 Center for Alcohol and Addiction Studies Department of Behavioral and Social Sciences Brown University School of Public Health Providence, RI United States

**Keywords:** smartphone, mHealth, smoking cessation, happiness, cigarettes

## Abstract

**Background:**

Nondaily smoking is an increasingly prevalent smoking pattern that poses substantial health risks.

**Objective:**

We tested the feasibility of using a smartphone app with positive psychology exercises to support smoking cessation in nondaily smokers.

**Methods:**

In this prospective, single-group pilot study, nondaily smokers (n=30) used version 1 of the *Smiling Instead of Smoking* (SiS) app for 3 weeks while undergoing a quit attempt. The app assigned daily happiness exercises, provided smoking cessation tools, and made smoking cessation information available. Participants answered surveys at baseline and 2, 6, 12, and 24 weeks after their chosen quit day and participated in structured user feedback sessions 2 weeks after their chosen quit day.

**Results:**

App usage during the prescribed 3 weeks of use was high, with an average 84% (25.2/30) of participants using the app on any given day. App use was largely driven by completing happiness exercises (73%, 22/30) of participants per day), which participants continued to complete even after the end of the prescribed period. At the end of prescribed use, 90% (27/30) of participants reported that the app had helped them during their quit attempt, primarily by reminding them to stay on track (83%, 25/30) and boosting their confidence to quit (80%, 24/30) and belief that quitting was worthwhile (80%, 24/30). Happiness exercises were rated more favorably than user-initiated smoking cessation tools, and 80% (24/30) of participants proactively expressed in interviews that they liked them. App functionality to engage social support was not well received. Functionality to deal with risky times was rated useful but was rarely used. Within-person changes from baseline to the end of prescribed use were observed for several theorized mechanisms of behavior change, all in the expected direction: confidence increased (on a 0-100 scale, internal cues: b=16.7, 95% CI 7.2 to 26.3, *P=*.001; external cues: b=15.8, 95% CI 5.4 to 26.1, *P=*.004), urge to smoke decreased (on a 1-7 scale, b=−0.8, 95% CI −1.3 to −0.3, *P=*.002), and perceptions of smoking became less positive (on a 1-5 scale, psychoactive benefits: b=−0.5, 95% CI −0.9 to −0.2, *P=*.006; pleasure: b=−0.4, 95% CI −0.7 to −0.01, *P=*.03; on a 0-100 scale, importance of pros of smoking: b=−11.3, 95% CI −18.9 to −3.8, *P=*.004). Self-reported abstinence rates were 40% (12/30) and 53% (16/30) of participants 2 and 24 weeks post quit, respectively, with 30% (9/30) biochemically validated as abstinent 2 weeks post quit.

**Conclusions:**

A smartphone app using happiness exercises to aid smoking cessation was well received by nondaily smokers. Given the high nonadherence and dropout rates for technology-delivered interventions reported in the literature, the high engagement with positive psychology exercises is noteworthy. Observed within-person changes and abstinence rates are promising and warrant further development of this app.

## Introduction

### Background

Cigarette smoking continues to be the leading cause of preventable disease and death in the United States, accounting for more than 480,000 deaths every year [1]. Although the prevalence of smoking has steadily declined over a number of years [2], an increasingly prevalent pattern of smoking is nondaily smoking. Currently, 24.3% of all adult smokers are nondaily smokers, which constitutes a 27% increase in prevalence in the last decade [3]. Nondaily smoking poses substantial health risks [4,5]. It is disproportionally represented in ethnic minority groups [6-10] and increasingly prevalent in adults with a mental health or substance use problem [11]. For smoking in general, substantial disparities continue to exist. In the general population, the prevalence of smoking is 15.5% [2]. This rate is substantially higher among American Indians and Alaska Natives (31.8%), persons with no more than a high school diploma/General Education Diploma (40.6%), persons living below the poverty level (25.3%), lesbian, gay, or bisexual adults (20.5%), and adults with serious psychological distress (35.8%) [2]. New patterns of smoking coupled with persisting disparities call for renewed efforts to provide easily accessible and engaging tobacco cessation support.

One promising emerging treatment option for providing such support is the use of smartphone apps. Smokers are motivated to quit smoking. Recent estimates suggest that 69% of all smokers want to quit; 52% made a quit attempt in the past year, but only 6% successfully quit [12]. Proven treatment strategies exist to support smoking cessation [13], increasing quit success rates from 5% in persons trying to quit smoking without support, to 16% with behavioral support, and 24% with combined behavioral and pharmacological support [14]. Existing treatments, however, are currently underutilized by smokers, with only 32% of current smokers having used counseling and/or medication when they tried to quit smoking [12]. Smartphone apps offer a way of providing behavioral counseling without the need to access the health care system or overcome logistical barriers to present for in-person counseling. They are particularly promising given the demonstrated effectiveness of text-messaging interventions to support smoking cessation [15]. Smartphone apps offer greater functionality than text-messaging interventions and thus potentially may be able to provide more engaging and effective means of providing smoking cessation support. Indeed, consumer interest in smoking cessation smartphone apps is high, with well over 200 apps in the Android store alone generating more than half a million downloads in 2014 [16]. The reach of smartphone apps is also excellent and increasingly equitable. Currently, 77% of US adults own a smartphone, with sharp upticks in lower income Americans and those aged 50 years and older [17]. In smokers motivated to quit smoking, smartphone ownership is particularly high (83%) [18], thereby making smartphone apps an increasingly equitable and viable option to aid smoking cessation.

Despite considerable interest in smoking cessation smartphone apps, evidence-based apps remain few and far between. Existing apps in the iPhone and Android stores generally fall short of adhering to clinical practice guidelines for smoking cessation [16,19,20] and underutilize functionality that would allow active engagement with smokers trying to quit [16]. Meanwhile, apps developed through research are rare and inaccessible. A recent systematic review identified only 6 smoking cessation apps with some level of scientific support, only 3 (50%) of which were available in an app store [21]. Thus, there continues to be a need for empirically grounded smoking cessation apps.

### Objectives

To address this need, we developed a smoking cessation app for nondaily smokers. Nondaily smokers are even less likely than daily smokers to seek or receive treatment [16]. To engage nondaily smokers in smoking cessation support, we chose a positive psychology approach, as detailed elsewhere [22], as the pursuit of happiness is generally appealing and nonstigmatizing and thus might overcome treatment resistance. Moreover, positive emotion during smoking cessation has been shown to increase an individual’s likelihood of successfully quitting smoking [23], and previous work has demonstrated the potential of positive psychotherapy to support smoking cessation [24,25].

This paper presents the findings of the first in a series of 3 studies designed to pilot-test and further develop the *Smiling Instead of Smoking* (SiS) app. In this study, 30 nondaily smokers interested in quitting smoking were asked to use the app (SiS1) for 3 weeks. We evaluated the feasibility and acceptability of the app on 3 dimensions: actual app usage patterns, direct feedback via survey and structured user feedback, and by testing if theorized within-person changes were taking place.

**Figure 1:**
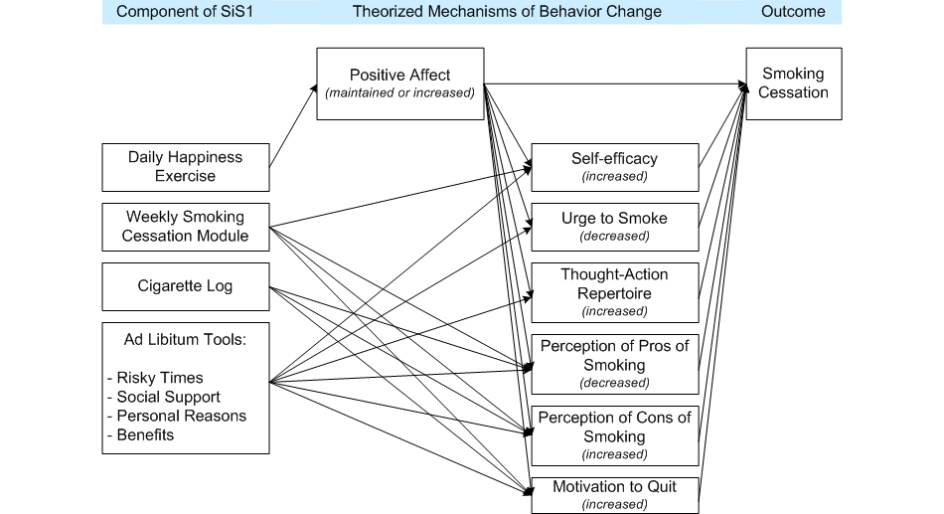
Conceptual model of how SiS1 was theorized to support smoking cessation.

On the basis of previous research, and as detailed in our previous paper detailing the design of this app [22], we hypothesized that engaging in positive psychology exercises would offset expected decreases in positive affect in the days leading up to the quit day [26], where lowered positive affect on the quit day has been linked to relapse [27,28]. We also hypothesized that engaging in positive psychology exercises would increase self-efficacy (ie, confidence to quit smoking and to stay quit) [29], reduce the desire to smoke [30,31], broaden the thought-action repertoire (ie, the number of alternative options for actions a person can come up with in a specific situation) to deal with challenging times [32], and decrease defensive processing of self-relevant health information (eg, the tendency to discount the importance of information that appears threatening or worrisome) [33], all of which should enable nondaily smokers to remain smoke-free after their chosen quit day. We operationalized processing of self-relevant health information by assessing 3 measurable aspects: the degree to which participants believed that smoking cigarettes would result in specific positive or negative effects, the importance they place on these effects, and their motivation to quit. The other components of SiS1 were designed to support these mechanisms, as summarized in Figure 1.

## Methods

### Participants

Participants were adult nondaily smokers, who were interested in using a smartphone app to help them quit smoking (recruited August 8, 2017-January 24, 2018). Study recruitment information was displayed on Craigslist, Smokefree.gov, a study recruitment website at Massachusetts General Hospital, websites of local universities, and an ad placed in a public transportation newspaper. Potential participants were included in the study if they were older than 18 years; smoked at least weekly, but no more than 25 out of the past 30 days; had a current quit intention; owned an Android smartphone (version 1 of the app was only created for the Android); spoke English; and were willing to come in for 2 in-person study visits. The study was approved by the Partners Healthcare Institutional Review Board. All participants provided informed consent.

### Procedure

Participants were phone-screened and asked to complete a screening test. To pass, participants had to (1) complete an Web-based baseline survey, and correctly (100%) respond to 5 randomly placed check-questions to verify that respondents were truly reading survey items; participants received US $10 versus US $35 for surveys with incorrectly versus correctly answered check-items, respectively, (2) provide contact information for 2 collaterals who would be able to assist research staff in locating participants for follow-ups, if necessary, and (3) provide their social security number to enable remuneration by check. Participants were notified by phone if they passed the screening test, and during this phone call, they were asked to set a quit day. An enrollment visit was then scheduled to occur 1 week before the chosen quit day at the research lab. During visit 1 (enrollment), participants were guided through downloading, installing, and using the app. After 3 weeks, participants returned for visit 2 (2 weeks after their chosen quit day, end of prescribed app support) for a structured user feedback session and the 2-week follow-up survey. Participants who reported abstinence provided a saliva sample for biochemical verification. Thereafter, participants completed follow-up surveys online 6, 12, and 24 weeks after their initially chosen quit day. All surveys were administered via the research electronic data capture system, REDCap, a secure, Web-based application designed to support data capture for research studies [34].

In total, we phone-screened 157 individuals, 58% (91/157) of whom signed online consent and took the baseline survey; 15% (24/157) decided against the study during the phone screen, and 27% (42/157) were ineligible, largely because of being daily smokers (n=25) or not owning an Android smartphone (n=12). After signing online consent, 33 individuals failed the check-items in the survey, and 27 changed their mind about participating in the study. A total of 31 individuals came in for visit 1; 1 person was unable to install the app because of too little phone storage space and decided against the study at that point. The remaining 30 comprise the sample reported in this paper. Remuneration was US $40 per survey and US $75 per in-person visit. Participants who missed a survey were contacted for subsequent surveys, unless participants actively withdrew from the study.

### Treatment

Participants received version 1 of the SiS app, as described elsewhere [22]. In brief, SiS version 1 is an Android smartphone app that engages participants in daily positive psychology exercises over the course of 3 weeks and provides behavioral support via 3 app-delivered sessions and ad libitum user-initiated tools. To engage participants in positive psychology exercises, each day the app selected 1 of 3 exercises at random, all of which have been previously shown to be effective in enhancing positive affect [35-37], and reminded participants to complete it, if they had not done so by 7 pm. The exercises were *3 Good Things* (participants enter text describing 3 good things that happened to them that day), *Savoring* (participants enter text describing 2 experiences they savored), and *Experiencing Kindness* (participants describe an act of kindness they performed and one they witnessed). The app logged all entries and allowed participants to browse through their log of happy moments.

Participants were also prompted to complete 3 app-delivered behavioral support sessions, scheduled to be completed 1 week before the chosen quit day, on the quit day, and 1 week after the quit day. Session content was based on recommended clinical guidelines [13], in that it asked about current smoking and smoking triggers, advised participants to quit smoking, assessed participants’ readiness to quit, addressed barriers they may perceive, assisted participants in setting a quit day, provided support during the quit attempt, and checked in with participants after their quit day. Tools were available to self-monitor cigarette use, specify personal reasons for quitting smoking, set personalized reminders to remain smoke-free during times of anticipated challenging times, enlist social support, display information on benefits of quitting smoking, and address commonly expressed concerns about quitting smoking. Participants were asked to use the app for 3 weeks, 1 week before and 2 weeks following their chosen quit day. Participants were free to continue using the app thereafter or to discontinue its use, as they deemed fit.

### Measures

#### App Usage

The app passively time stamped interactions with the app, from which we calculated the percentage of participants who used each function on a given day.

#### User Feedback

##### Via Survey

In the 2-week survey, participants were asked to rate the ease-of-use and usefulness of each component of the app (10 items each) on a 4-point Likert scale (ease-of-use: 0=*not easy at all*, 1=*somewhat easy to use*, 2=*easy to use*, and 3=*very easy to use*; useful: 0=*not at all useful*, 1=*somewhat useful*, 2=*usef* ul, and 3=*very useful*). Participants also indicated if the app helped them in their quit attempt (*yes/no*) and in which ways the app helped them (10 items, rated on a 5-point Likert scale, 1=*strongly disagree*, 2=*disagree*, 3=*neither agree nor disagree*, 4=*agree*, and 5=*strongly agree*).

##### Via Structured User Feedback Session

During visit 2, participants were presented with a summary of their interactions with the app. Staff asked 22 specifics on phenomenology of nondaily smoking, specific suggestions for adding to drop-down menus, feedback about specific tools in the app, and participants’ bottom-line take-home recommendation for adding or removing features from the app.

#### Indices of Putative Mechanisms of Behavior Change

Surveys assessed constructs theorized to underlie the process of smoking cessation. For ease of interpretation, we calculated scale scores by averaging across items (ie, rather than sum scoring), so that scores can be interpreted directly on the scale participants used to rate them. Baseline Cronbach alphas observed in this study are reported below.

##### Positive Affect

The Positive and Negative Affect Schedule (PANAS, 10 items, 1=*very slightly or not at all*, 5=*extremely*) [38] uses mood adjectives to assess to what extent participants felt specific emotions during the past week. Participants also used a single-item slider (0=*not at all happy,* 100=*extremely happy*) to indicate how happy they were feeling right before completing the survey. Overall satisfaction with life and happiness were assessed with the Satisfaction with Life Scale (5 items, 1=*strongly disagree*, 7=*strongly agree*) [39] and the Subjective Happiness Scale (4 items, item-specific anchor points, eg, *In general, I consider myself*…,1=*not a very happy person*, 7=*a very happy person*) [40].

##### Self-Efficacy

The Smoking Self-Efficacy Questionnaire (24 items; 0=*not at all confident*, 100=*extremely confident*) [41] assessed confidence in the ability to abstain from smoking when facing internal (eg, feeling depressed) and external stimuli (eg, being with smokers).

##### Urge to Smoke

The brief Questionnaire of Smoking Urges (10 items, 1=*strongly disagree* and 7=*strongly agree*) [42] assessed craving (eg, *I have an urge for a cigarette*).

##### Breadth of Thought-Action Repertoire

To measure the breadth of participant’s thought-action repertoire, we used the Twenty Statements Test [43]. In this test, participants are asked to describe a strong emotion they have just experienced, take a moment to feel it deeply, and are then instructed: *Given this feeling, please list all the things you would like to do right now*. This instruction is followed by 20 blank lines that began with the following: *I would like to…* To score, the number of items completed is counted, resulting in a score from 0 to 20, with larger scores indicating a larger thought-action repertoire. In this study, participants were asked to *name the strongest emotion you feel when thinking about your quit attempt.*

##### Perception of Pros and Cons of Smoking

The Attitudes Towards Smoking Scale-18 items (1=*strongly disagree*, 5=*strongly agree*) [44] assessed the degree to which participants perceive adverse effects of smoking (eg, *smoking is ruining my health*), psychoactive benefits of smoking (eg, *smoking calms me down when I am upset*), and pleasure of smoking (eg, *it feels so good to smoke*). The Decisional Balance Inventory for Smoking (6 items, 0=*not at all important* and 100=*extremely important*) [45] assessed perceived importance of commonly expressed pros (eg, *Smoking cigarettes relieves tension*) and cons (eg, *My cigarette smoking bothers other people*). Participants also used single-item sliders (0=*not at all important*, 100=*extremely important)* to rate their own defined pros and cons (eg, *Think about all the things you LIKE/LOVE about quitting/being smoke-free. Taken together, how important are those things to you RIGHT NOW*?).

##### Motivation to Quit Smoking

The Commitment to Quitting Smoking Scale (8 items, 1=*strongly disagree*, 5=*strongly agree*) [46] captures the extent to which persons feel personally bound or obligated to persist in quitting smoking despite potential difficulties, craving, and discomfort. Participants also used a single-item measure (0=*not motivated at all*, 100=*extremely motivated*) to rate their motivation (ie, *How MOTIVATED are you to quit smoking/stay quit?*).

#### Outcome of the Smiling Instead of Smoking–Supported Quit Attempt

##### Abstinence

In each survey, participants were asked: *What is your smoking status?* The response options were *I smoke daily*, *I smoke nondaily*, and *I do not smoke at all*. If participants reported not smoking, they were asked *Have you been abstinent for 7 days?* (*yes/no*). If yes, they were asked *Have you been abstinent for 30 days?* (*yes/no*). For the primary endpoint (ie, 2 weeks post quit), abstinence self-reports were biochemically verified using saliva cotinine (<15 ng/mL) [47,48].

### Analytic Strategy

To describe feasibility, acceptability, and outcome, we calculated descriptive statistics. For user feedback sessions, content analyses were performed on the responses to the 22 questions by 2 independent coders. Major themes were identified. Coding differences were reviewed with the study team to resolve discrepancies.

To test if nondaily smokers using SiS experienced changes over time on constructs theorized to underlie smoking cessation, we used the online survey data (n=30) and fit one repeated measures mixed effects model per construct of interest, where the construct was the dependent variable and time (baseline, 2, 6, 12, and 24 weeks) was the predictor. Per protocol, the primary endpoint of interest was treatment end (ie, 2 weeks after the chosen quit date). Correlations over time were modeled with an unstructured covariance matrix. Given the explorative nature of this study, we did not correct for multiple testing.

## Results

### Participant Characteristics

The participants’ age ranged from 23 to 63 years, and the mean age was 45 years (SD 14.1). Most participants were male (22/30, 73%; Table 1). Our sample matched published rates of nondaily smoking [49] and quit intentions [7] but had greater racial-ethnic diversity than is nationally representative: 40% (12/30) of our participants were non-Hispanic white; 55% of nondaily smokers were non-Hispanic white in the National Health Interview Survey 2015 [50]. Nearly all participants completed all surveys; completion rates were 100% (30/30), 93% (28/30), 97% (29/30), and 97% (29/30) at follow-ups occurring 2, 6, 12, and 24 weeks after the quit day, respectively.

### App Usage

**Figure 2:**
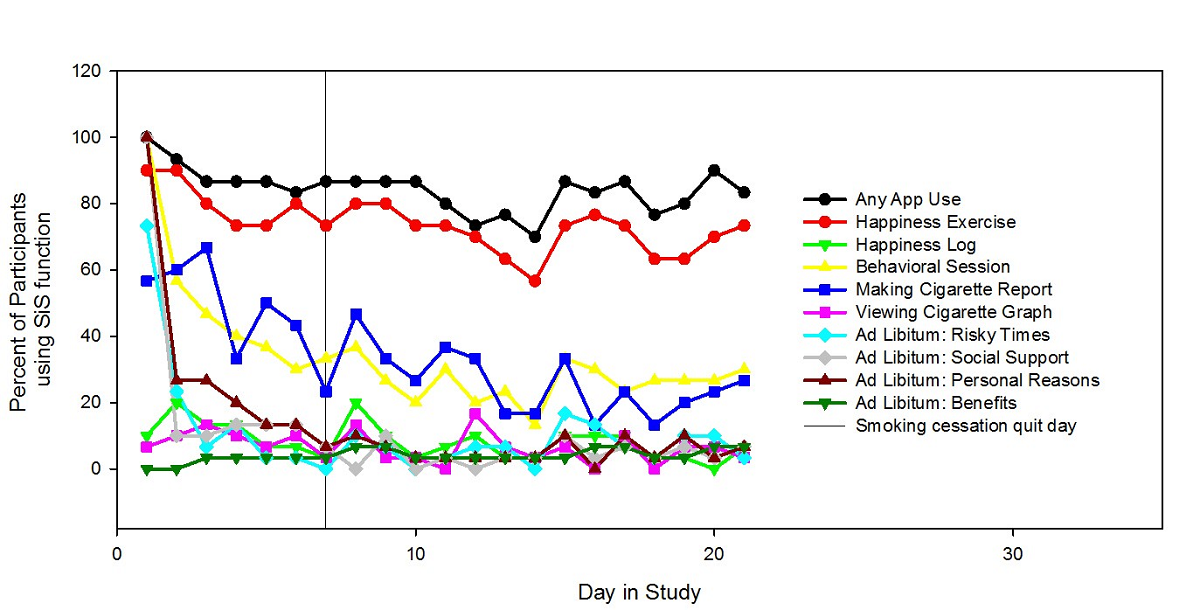
App usage during the prescribed 21-day period. SiS: Smiling Instead of Smoking.

App usage during the prescribed 21 days of use was high (Figure 2). On average, and excluding the first day on which participants were guided through the app, 84% (25.2/30) of participants used the app on any given day. App use was primarily driven by the completion of the daily happiness exercises (73%, 22/30) of participants on any given day). Viewing the happiness log occurred relatively rarely, with only 6% (2/30) of participants doing so on a given day. This usage pattern, however, was in line with viewing the graph generated by logging one’s cigarettes (7%, 2/30) and using other ad libitum functions, such as *Risky Times* (7%, 2/30), *Social Support* (7%, 2/30), *Personal Reasons* (10%, 3/30), and *Benefits* (3%, 1/30).

**Figure 3:**
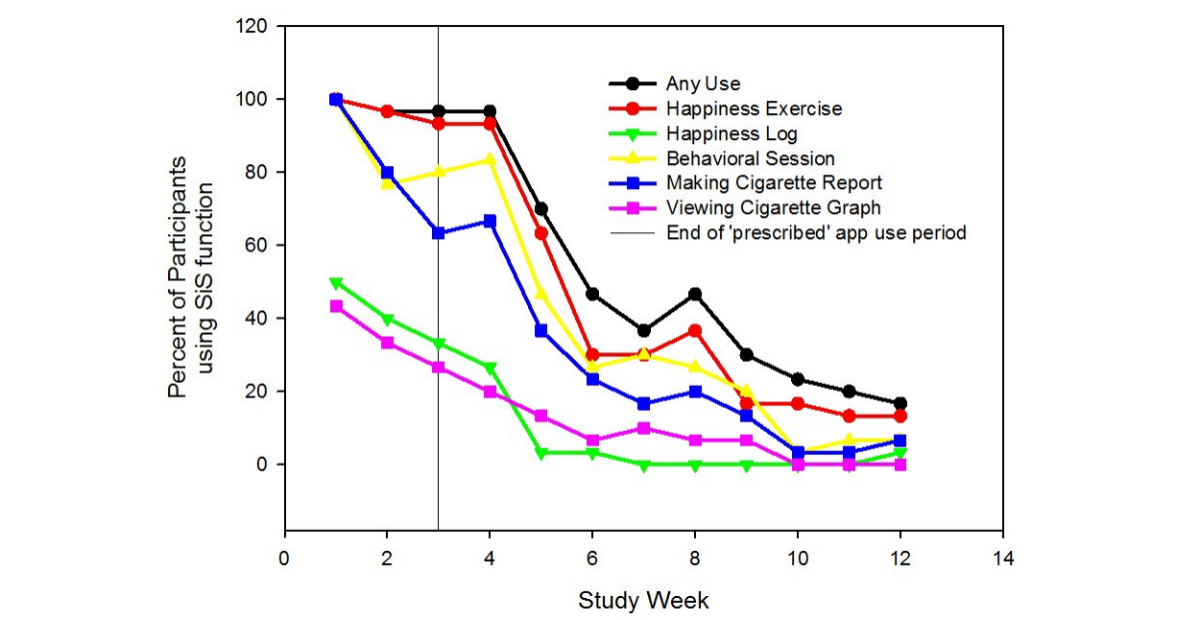
App usage per study week. SiS: Smiling Instead of Smoking.

In addition, frequently used were the behavioral sessions, which were intended to be accessed once per week but could be accessed multiple times, and making cigarette reports in the cigarette log. Within a weekly context (Figure 3), on average, 86% (25.8/30) of participants completed behavioral sessions during the prescribed 3 weeks, and 34% (10.2/30) made smoking reports, where it was expected that nondaily smokers would stop making smoking reports as they quit smoking; 97% (29/30) of participants completed happiness exercises in any given week during the prescribed 3 weeks.

**Table 1:** Sample characteristics (n=30).

Characteristics			Values
**Demographics**			
	Age (years), mean (SD)		44.7 (14.1)
	Gender (female), n (%)		8 (27)
	**Race, n (%)**		
		White	12 (40)
		Black	13 (43)
		Other or unknown	5 (17)
	Hispanic, n (%)		2 (7)
	**Education, n (%)**		
		High school or less	8 (27)
		Some college	11 (37)
		Bachelor’s or higher	11 (37)
**Smoking characteristics**			
	Days smoked in past 30 days, mean (SD)		15.6 (6.0)
	Cigarettes smoked per smoking day, mean (SD)		4.5 (2.9)
	Ever smoked daily? Yes, n (%)		18 (60)
	Ever quit before? Yes, n (%)		21 (70)

After the end of treatment, some participants continued to use the app. Excluding week 4, during which participants may still have been completing visit 2, 40% (12/30) of participants completed happiness exercises during any given week from week 5 to 8.

### User Feedback

Survey responses indicated that the best-rated features, both in terms of ease-of-use and usefulness, were *Scheduling Your Quit Day* and *Your Reasons for Quitting* (Table 2). Nearly all participants (90%, 27/30) felt that the app had helped them in their quit attempt (Table 3). In particular, the app appeared to reinforce the value of quitting, with ratings of 4.1 (on a 1-5 scale) for *reminded me why I wanted to quit* and 4.0 for *made me think it was worthwhile for me to quit*. The app’s role in reminding participants to *stay on track* (83%, 25/30 indicated agreement) and in giving confidence (80%, 24/30 indicated agreement) were also important aspects. The app was less successful in helping participants seek social support (57%, 17/30 endorsement) and dealing with risky situations (57%, 17/30 endorsement; Table 3).

**Table 2:** Ratings of the Smiling Instead of Smoking functions.

Function		Ease of use^a^, mean (SD)	Useful^b^, mean (SD)
**Completing the positive psychology exercises every day**		2.0 (0.9)	1.8 (0.9)
	Specifically, completing *3 Good Things*	2.1 (0.9)	1.9 (0.8)
	Specifically, completing *Savoring*	2.1 (0.8)	1.9 (0.9)
	Specifically, completing *Experiencing Kindness*	2.1 (0.9)	1.9 (0.9)
Completing the smoking sessions		2.0 (0.8)	1.8 (0.9)
**Accessing and updating your smoking cessation tools**		1.8 (0.9)	1.7 (1.0)
	Specifically, Scheduling Your Quit Day	2.3 (0.9)	2.1 (0.9)
	Specifically, Your Reasons for Quitting	2.4 (0.7)	2.3 (0.7)
	Specifically, Managing Your Challenging Times	1.9 (0.8)	1.6 (0.9)
	Specifically, Enlisting Your Social Support	2.0 (0.8)	1.6 (1.0)

^a^Ease of use was rated on a 4-point scale: 0=*not easy at all*, 1=*somewhat easy to use*, 2=*easy to use*, and 3=*very easy to use*.

^b^Useful was rated on a 4-point scale: 0=*not at all useful*, 1=*somewhat useful*, 2=*useful*, and 3=*very useful*.

**Table 3:** Perceptions of how the app might have helped.

Perceptions	Mean^a^ (SD)	Agree, n (%)
The app helped remind me to stay on track with quitting.	3.9 (0.9)	25 (83)
The app gave me confidence that I can quit smoking.	3.9 (1.0)	24 (80)
The app made me think that it was worthwhile for me to quit.	4.0 (1.1)	24 (80)
The app made me feel that someone cared if I quit.	3.9 (1.1)	23 (77)
The app reminded me why I wanted to quit.	4.1 (0.8)	23 (77)
The app helped me stay positive while quitting.	3.8 (1.1)	21 (70)
The app gave me the feeling I could get trusted advice at any time.	3.7 (1.1)	21 (70)
The app made me feel that I knew the right steps to take to quit.	3.5 (1.1)	18 (60)
The app motivated me to reach out to the people in my life about quitting.	3.4 (1.2)	17 (57)
The app helped me deal with risky smoking times.	3.5 (1.2)	17 (57)
Taken altogether, do you think that the app helped you in your quit attempt?	—^b^	27 (90)

^a^Rated on a 5-point Likert scale: 1=*strongly disagree*, 2=*disagree*, 3=*neither agree nor disagree*, 4=*agree*, 5=*strongly agree.*

^b^Not applicable.

**Figure 4:**
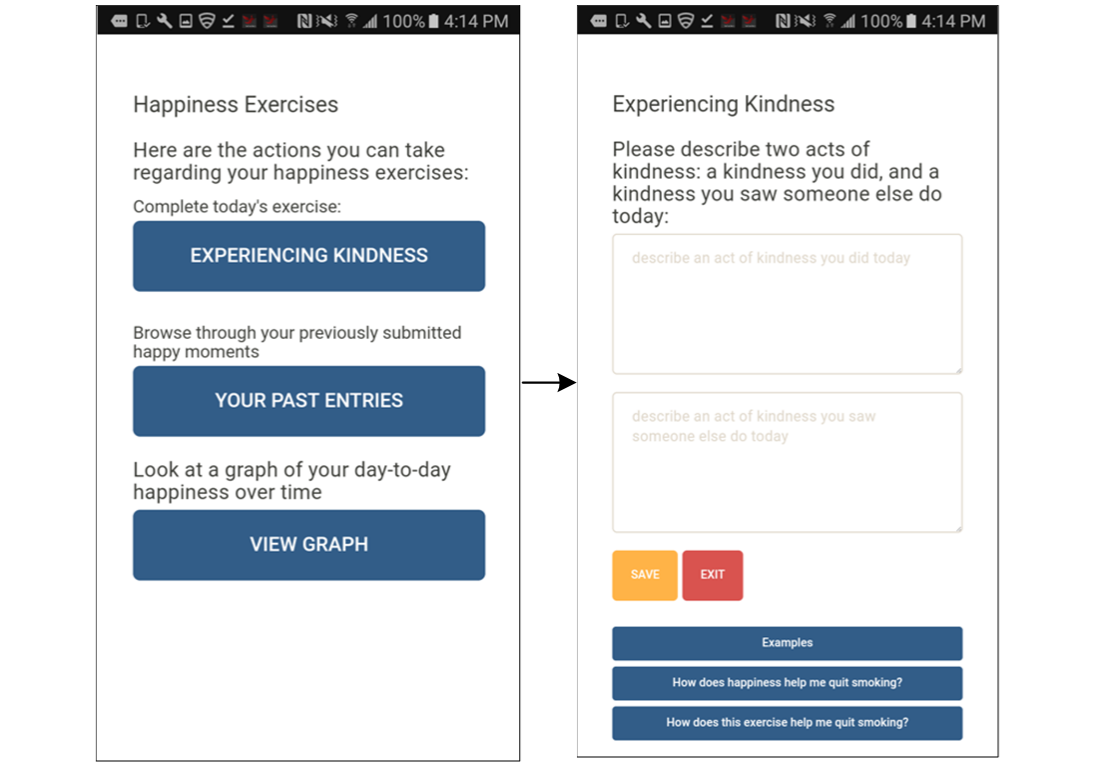
Screenshots of completing the daily happiness exercises in SiS1. SiS: Smiling Instead of Smoking.

**Figure 5:**
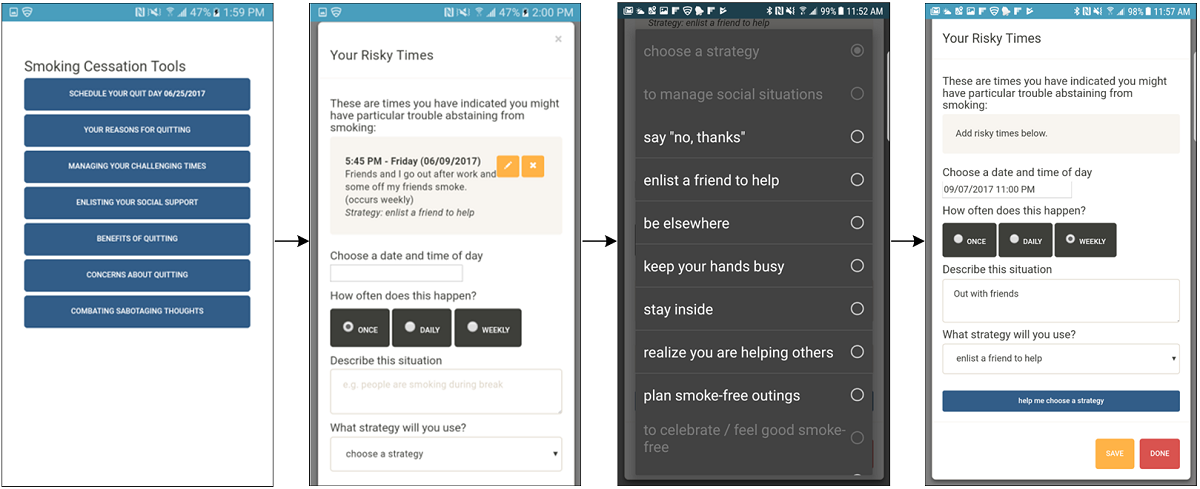
Screenshots of completing the ad libitum tool Risky Times in SiS1. SiS: Smiling Instead of Smoking.

During structured user feedback sessions, 80% (24/30) of participants expressed that they liked the happiness exercises (Figure 4) and felt it was good for them to complete them. Participants (23%, 7/30) wanted more happiness exercises to provide greater variety. Regarding behavioral counseling, participants (47%, 14/30) liked the content but expressed a desire for shorter, more frequent, proactive (ie, initiated by the app) interactions that are less wordy and use more graphics. Participants highlighted problems with the *Risky Times* and *Social Support* functions (Figure 5), with many (33%, 10/30) expressing that they found the *Risky Times* tool useful but too cumbersome, and most (43%, 13/30) disliking the functionality for enlisting the help of social support. Several participants (13%, 4/30) reacted negatively to the suggestion that supportive people in their lives could *reward them for reaching smoking cessation milestones*. Rather than involving people in their lives in their smoking cessation process, participants suggested adding links to or information about support groups. If involving people in their lives, participants suggested that it would be better to do so without a focus on smoking cessation (eg, meeting up to go running).

When asked about their personal bottomline recommendation for the app, more than half of the participants (67%, 20/30) did not want to cut anything from it. At the same time, most participants (83%, 25/30) felt that things could be added to the app, where the most frequent recommendations were to add functionality to encourage more frequent interaction with the app (37%, 11/30), reinforce the pros of quitting (20%, 6/30), add a component to interact with other app users (17%, 5/30), add gamification to the app (10%, 3/30), and/or a relational agent (10%, 3/30).

### Indices of Putative Mechanisms of Behavior Change

Within-person changes from baseline (ie, as measured in the survey administered as part of the screening procedure before study enrollment) to treatment end (ie, 2 weeks after the chosen quit day) were observed for several theorized mechanisms of behavior change, all in the expected direction (Table 4). Namely, on average, participants indicated greater confidence in their ability to quit smoking and stay quit, both in response to internal and external cues, and less desire to smoke, including reduced positive outcome expectancies of smoking, as measured by multiple scales (ie, pleasure of smoking, psychoactive benefits of smoking, personal importance of pros of smoking).

After the end of treatment, these effects were sustained through study end (ie, 6 months after the chosen quit day), with the exception of decreases in *pleasure of smoking*, as measured via the ATS, which did not reach statistical significance at the 6- or 12-week assessment (*P=*.06 for both tests, comparing 6-week/12-week to baseline), but were statistically significant 24 weeks after the quit day (b=−0.54, *P=*.009). At the same time, however, other trends emerged that were less supportive of smoking cessation. Namely, the importance of the pros of quitting, as measured via a single item, was significantly lower 6, 12, and 24 weeks after the quit day by 10 to 14 points on a 0 to 100 scale (*P=*.02, .02, and .01, respectively) than that at baseline. Similarly, motivation decreased and was significantly lower than baseline 6 months after the quit day, by about 14 points (*P=*.03). The thought-action repertoire, as measured by the TST, also decreased after treatment end (*P=*.008, .006, and .01, respectively, at 6, 12, and 24 post quit, compared with baseline). Similarly, positive affect was lowered, with a significantly lower past week PANAS score 12 weeks post quit (b=−0.4, *P=*.004) and a significantly lower momentary happiness score 6 weeks post quit (b=−10.3, *P=*.04).

### Outcome of the Smiling Instead of Smoking–Supported Quit Attempt

At the end of prescribed app use, 40% (12/30) of participants reported having been abstinent for the past 7 days. Salivary cotinine values for 3 participants, however, were greater than 15 ng/mL without having reported current use of other nicotine products (ie, e-cigarette use and nicotine replacement therapy). Thus, the biochemically verified point-prevalence abstinence rate was 30% (9/30). Following the end of prescribed app use, and assuming that survey nonresponders were not abstinent, 7-day point-prevalence abstinence was self-reported by 53% (16/29), 48% (14/29), and 55% (14/29) of participants 6, 12, and 24 weeks after the quit day, respectively; 30-day point-prevalence abstinence was self-reported by 41% (12/29), 45% (13/29), and 48% (14/29) of participants 6, 12, and 24 weeks after the quit day, respectively.

**Table 4:** Within-person changes on theorized mechanisms of change from baseline to the end of treatment.

Construct, scale		Cronbach alpha at baseline	Baseline^a^, mean (SD)	Scale range	2-week versus baseline	
					b^b^ (CI)	*P* value
**Happiness**						
	PANAS^c^ (past week positive affect)	.88	3.6 (0.6)	1-5	−0.2 (−0.4 to 0.1)	.17
	PANAS (past week negative affect)	.83	1.7 (0.5)	1-5	0.1 (−0.1 to 0.4)	.27
	Single item—how happy right now	—^d^	69.9 (17.8)	0-100	−6.0 (−13.9 to 1.9)	.13
	Satisfaction with Life	.81	4.0 (1.2)	1-7	0.2 (−0.2 to 0.6)	.36
	Subjective Happiness	.88	5.4 (1.3)	1-7	−0.1 (−0.5 to 0.3)	.52
**Self-efficacy**						
	SEQ-12^e^ (internal cues)	.92	44.9 (24.3)	0-100	16.7 (7.2 to 26.3)	.001^f^
	SEQ-12 (external cues)	.88	47.3 (25.3)	0-100	15.8 (5.4 to 26.1)	.004^f^
**Desire**						
	Questionnaire of Smoking Urges (smoking urges)	.86	3.1 (1.1)	1-7	−0.8 (−1.3 to −0.3)	.002^f^
**Breadth of thought-action repertoire**						
	Twenty Statement Test	—	9.5 (6.8)	0-20	−2.5 (−5.2 to 0.3)	.08
**Processing self-relevant health information**						
	ATS^g^ (adverse effects)	.94	4.2 (0.9)	1-5	0.0 (−0.3 to 0.4)	.86
	ATS (psychoactive benefits)	.81	3.6 (0.8)	1-5	−0.5 (−0.9 to −0.2)	.006^f^
	ATS (pleasure)	.88	3.0 (1.0)	1-5	−0.4 (−0.7 to −0.0)	.03^h^
	DCB^i^ (importance of pros of smoking)	.64	48.6 (21.8)	0-100	−11.3 (−18.9 to −3.8)	.004^f^
	DCB (importance of cons of smoking)	.64	60.6 (25.6)	0-100	−4.3 (−13.8 to 5.2)	.37
	Single item—pros of quitting	—	92.3 (11.0)	0-100	−6.6 (−14.7 to 1.5)	.11
	Single item—cons of quitting	—	50.4 (39.9)	0-100	2.0 (−16.8 to 20.7)	.83
	Commitment to Quitting Smoking Scale (commitment to quitting)	.91	3.8 (0.7)	1-5	−0.2 (−0.5 to 0.2)	.35
	Single item—how motivated	—	88.0 (13.7)	0-100	−5.1 (−12.6 to 2.4)	.18

^a^Baseline occurred before SiS download, and 2-week follow-up occurred at the end of the prescribed 21 days of app use (ie, 2 weeks post quit day).

^b^Repeated measures mixed effects model parameter estimate.

^c^PANAS: Positive and Negative Affect Schedule.

^d^Not applicable.

^e^SEQ: Self-Efficacy Questionnaire.

^f^Flags differences with *P*<.01.

^g^ATS: Attitudes Towards Smoking.

^h^Flags differences with *P*<.05.

^i^DCB: Decisional Balance Inventory for Smoking.

## Discussion

### Principal Findings

In this first pilot test of version 1 of our smoking cessation app *SiS*, we evaluated the feasibility and acceptability of the app. To provide a comprehensive test, we used 3 types of data sources: passively recorded app usage, standardized survey self-reports, and face-to-face feedback sessions with participants after they had used the app.

On the whole, the gathered data support the notion that leveraging positive psychology tools, or more precisely, happiness exercises, may be well received by nondaily smokers and feasible, at least over the short term. A very high percentage of participants completed the daily exercises throughout the prescribed period of app use, and after completing them for 3 weeks, participants rated the exercises as easy to use and useful. Moreover, in structured user feedback sessions, participants pointed out that they enjoyed doing the happiness exercises, and many continued to complete them long after the formal treatment period was over. As was our goal, by the end of prescribed app use, positive affect appears to have been maintained at precessation levels; at least, we did not observe any statistically significant declines by treatment end, where, of course, our ability to detect such differences was limited because of our small sample size. Once prescribed app use had ended, there was some evidence of a decline in the positive affect.

In designing the app, we had hypothesized that maintained positive affect would favorably affect known mechanisms of smoking cessation [22]. In line with these expectations (though certainly not a direct test thereof), we observed within-person changes such as increased confidence in ability to quit smoking, decreased smoking urges, and a shift to a less positive view of smoking. All of these changes are changes that are hoped for in successful smoking cessation. In particular, an increase in confidence is an important mechanism by which benefit is conferred in text-messaging approaches to smoking cessation [51], making the observed increases in users of the SiS app a promising sign of its potential effectiveness.

Also promising were self-reported quit rates. To date, mobile health (mHealth) approaches to smoking cessation have only been tested in daily smokers. Here, abstinence rates have ranged from 26% to 36%, with 26% of daily smokers using the app *Clickotine* (n=416) reporting being 30 days abstinent at 2-month follow-up [52]; 36% of daily smokers using an mHealth program combining real-time tailored advice with asynchronous secure messaging with a cessation counselor reporting abstinence 5-month follow-up [53]; and an average of 28% (treatment) versus 13% (control) of daily smokers across 4 randomized controlled trials testing text-messaging smoking cessation interventions reporting abstinence 4 to 6 weeks post quit [54-57]. Abstinence rates in our study were consistently higher (ie, ≥40% [≥12/30] at 6, 12, and 24 weeks post quit).

### Areas for Further Development

These promising abstinence rates notwithstanding, our data also suggested that it may be useful to consider implementing more app-initiated, varied, and frequent interactions with the app, and to do so over a longer period. This theme emerged from several types of data. First, participants directly told us that they desired more app-initiated, frequent, varied interactions with the app. Second, once app support was withdrawn, some less-than-favorable within-person changes emerged, where there was evidence of sporadically lowered positive affect, decreased motivation, and diminished thought-action repertoire. Although these decreases did not seem to impact smoking cessation in this small pilot sample of engaged and motivated study participants, they may translate to poorer outcomes in smokers who use the app on their own without research staff interactions. This is especially of concern given that participants indicated in surveys that a key reason the app helped them was because it reminded them to stay on track. As an app can only serve as a reminder when users are actively engaging with it, we think it may be important to continuously engage users with the app. This feedback is in line with emerging findings on adherence to technology-delivered interventions. Dropout and nonadherence are often high in technologically delivered interventions [58], which may be problematic, because higher adherence is associated with better mental health outcomes [59,60]. Moreover, recent findings suggest that adherence to technology-delivered interventions can be improved through frequent intended usage [61]. Identifying the optimal level of app engagement to promote smoking cessation and other health behaviors is an important topic for future research.

The happiness exercises seemed to engage persons well on a day-to-day basis and indeed, were the primary driver of interactions with the app (Figure 2). Nevertheless, doing the same 3 exercises can become tiresome over a longer period, and participants indicated their desire for additional types of exercises to be included in the rotation. Participants also indicated that they liked the content provided in the behavioral counseling sessions, and, even though only offered on a weekly basis, this content was an important contributor to overall app use. To support more frequent app interaction, it may be useful to present this content in shorter, more frequent installments.

Another theme for improvement that emerged was an increased focus on the pros of quitting smoking. Participants recommended that we add more content on the pros of quitting smoking to future versions of the app. This recommendation was supported by the quantitative data on within-person changes, where the importance of the pros of quitting diminished after treatment end, when app support was withdrawn. SiS version 1 included functionality that asked participants to list their personal reasons for quitting smoking during the first behavioral counseling session, a list that could be updated by users subsequently. This function was, surprisingly, the highest user-rated function of the app (Table 3). Users of the SiS app used the *Personal Reasons* functionality only sporadically, possibly because this functionality did not offer novelty over time. Future studies should address how to improve user engagement with more varied content about the pros of quitting smoking over a longer period.

Furthermore, a noteworthy aspect was the negative appraisal of the social support functionality in the SiS app. We had included app functionality that encouraged app users to reach out to important people in their lives to support them in their quit attempts because enlisting social support has been recommended for smoking cessation support [13]. In retrospect, however, this functionality was ill chosen because nondaily smokers oftentimes keep their smoking hidden from important people in their lives and frequently will deny their smoking habit when asked by family, friends, and health care providers [62]. Other ways to encourage the attainment of social support, such as, for example, anonymous online chat groups and other social media technologies [63,64], may be more suitable for nondaily smokers.

### Limitations

First, as a first pilot study of an evidence-based smoking cessation app that leverages positive psychology exercises to engage and support nondaily smokers in their quit attempts, this study is subject to several important limitations, with the chief limitation among them being the small sample size and the lack of a randomized control group, which preclude conclusions about the potential efficacy of the app. Observed effects are likely influenced by a self-selection process, where participants particularly interested in using a smoking cessation app, and thus potentially particularly likely to benefit from such an app, enrolled in the study. Second, a surprisingly small percentage of women participated in the study. In a similar study with identical eligibility and screening procedures, with the exception of no requirement to attend in-person visits [65], we recruited 66% women, suggesting that perhaps the in-person component was a deterrent. The requirement to provide the social security number information to allow us to provide remuneration was generally not seen as a deterrent, with only 1 of 157 screened potential participants deciding against the study because of it. Finally, it must be noted that biochemical verification of smoking status was only conducted once, at the primary endpoint and not at subsequent follow-ups, and only used salivary cotinine and not carbon monoxide in exhaled breath. Smoking status was also not reassessed at enrollment, and participants may have changed their smoking in between screening and enrollment, which were on average 15±13 days apart.

### Conclusions

This first feasibility test of a smoking cessation app for nondaily smokers shows that an app that leverages positive psychology exercises to engage and support users is well received by nondaily smokers, who used the app on a near daily basis for the prescribed 3 weeks. Users of the app appeared to make important smoking cessation progress, showed within-person changes on several theorized mechanisms of change, and proactively expressed liking the positive psychology approach of the app. Further development of this line of work is warranted.

## Abbreviations

ATS: Attitudes Towards Smoking
DCB: Decisional Balance Inventory for Smoking
PANAS: Positive and Negative Affect Schedule
SEQ: Self-Efficacy Questionnaire
SiS: Smiling Instead of Smoking
